# Recent Applications of Electricity

**Published:** 1902-11-08

**Authors:** George Parker

**Affiliations:** Assistant Physician Bristol General Hospital


### Hospital Clinics and Medical Progress.

### RECENT APPLICATIONS OF ELECTRICITY.

By George Parker, M.A., M.D.Camb., Assistant Physician Bristol General Hospital.

The use of electricity in medicine has been greatly increased during the last few years, partly from the advance of electrical science and the invention of new instruments or the improvement of old ones, and partly from the application of electricity to purposes for which its value was till lately unknown. The discovery of the Roentgen rays and their activity in skin affections and on certain kinds of new growths, as well as their utility for purposes of diagnosis is not the only direction in which progress has been made. High frequency currents and improved machines for static electricity have opened up new fields for investigation; and the extended use of electric light has given us not only a substitute for hand batteries, but has practically resulted in the use of a new form of current, the sinusoidal, for a great number of therapeutic purposes, with results that compare favourably with those of the old taradic and galvanic currents.

So much discredit has been thrown on the subject from the unfounded pretensions of quacks, and from our ignorance of much of the real action of electricity on the body, that we are apt to forget the proved value of electricity for certain definite purposes. A ready answer to such doubts is afforded by the number of cases of lupus and rodent ulcer rapidly cured by the Roentgen rays which are now to be seen everywhere; the quick recovery of many forms of peripheral paralysis, and the relief of lumbago and various painful joint affections by electrical treatment. Moreover any theoretical doubt as to its influence on the growths of wasted muscles are set at rest by the results of Debedat's experiments in which the effects produced by the current were carefully measured and weighed, so as to give for almost the first time a scientific basis for the claims which have been made. Debedat[1] tested certain young animals, applying to the hamstring muscles of the left leg various forms of electric current for four minutes daily for 20 days, while the right leg was left untouched in each case as a control. At the end of this time the animals were killed and the muscles of each leg were weighed separately. It was found that with (*a*) the Faradic current applied for one second, followed by rest for one second during the four minutes, there was a gain of weight of 40 per cent. in the muscles of the limb treated over the untreated one; (*b*) a galvanic current applied with the same intermissions gave a gain of 18 per cent.; (*c*) a galvanic current applied without intermissions gave only a slight increase over the other limb, and caused adhesions to form between the skin and the muscles; (*d*) a Faradic current without periods of rest caused an actual loss; (*e*) finally, the static spark applied every other second gave no effect at all.

Of course these results only bear upon one of the uses of electricity, viz., its influence on nutrition, nor do they show how the effect is produced, whether it be by a direct stimulus of assimilation or by growth due to increased exercise. Similarly, the more rapid recovery (in cases of bilateral paralysis) of the limb which has been treated by electricity affords a sound reason for treatment, though it does not explain the nature of the curative action. Clearly, Debedat's results are not due to direct suggestion, to which four-fifths of electrical cures have been ascribed, and they show that Duchesne's advocacy of the induced current was not groundless. Of late years there has been a tendency to ascribe nutritional effects only to the galvanic current, but from the practical point of view this can no longer be maintained.

It is to be wished that writers upon electricity had built upon the solid basis of facts like these instead of expressing pious opinions as to the general improvement they believed to be due to it. Every course of electricity should, if possible, be given with exact measurements of the current applied, its amperage, interruptions and duration, and the results as shown by altered reactions and bulk of muscles or by the dynamometer. Writers on psychophysics have devised many mechanical tests which may be of use if only to disprove the absurd claims of the ignorant. When systemic effects are desired, the results as shown by accurate estimations of the blood, urine, respiration and heart's action can alone be depended upon. Some few facts of this kind have been collected with regard to the static and high frequency currents as well as with the older forms, but from their scantiness hardly any important lessons have as yet been learnt. It may be added that magnetism appears to have no effect whatever on any physiological process.

In view of the practical difficulty of getting a thoroughly insulated bath, a very satisfactory way of avoiding all danger from earth currents or leakage is to work entirely from a secondary circuit. The current from the main, say at 105 volts is sent through a substantially made induction apparatus and induces in a secondary coil a fresh current of the same form, the voltage of which is to the voltage of the primary current in direct proportion to the number of turns of the wire. Thus it may be reduced to 5, 10, or 20 volts, the largest required even for the electric bath. Moreover, as this current begins and ends in the secondary coil there is no danger from leakage to earth and the patient can suffer no harm if the insulation of the bath is incomplete. This small and manageable current is raised or lowered within the limits of its 20 volts with the greatest smoothness by a sliding rheostat.

We may even use in default of any special apparatus the sledge coil of an ordinary Faradic battery. A lamp of 8 or 16 candle-power is interposed between the wall plug and the sledge to reduce the current, and the hammer is screwed down tight. In this way a secondary current of the sinusoidal type and moderate voltage is obtained at the binding screws marked s, and the strength can be regulated by sliding the sledge in the ordinary manner. Thus it is possible to employ the alternating current from the main in the consulting-room or the patient's house without expensive instruments. Care should be taken, however, that sufficient lamp resistance is interposed between the wall plug and the sledge coil, or else the latter may be fused or damaged. Instead of the ordinary electrodes two basins of water or at least large wet sponges are necessary. With a small wooden wash-tub a most agreeable and effective electric sitz bath can be given in cases of lumbago and sciatica. The ends of the wires may be fastened to strips of copper and hung inside the tub, care being taken that they reach below the surface of the water and do not touch the patient. Metal baths are difficult to use, and even if japanned the results are very uncertain, so that earthenware or wood can alone be recommended. The chief point to remember is that care must be used not to touch the apparatus in the region of the primary current, *e.g.*, the lamp wires, or near the hammer, lest we get a shock from the primary current.

Some of my nurses who were standing on a wet floor were much astonished to get a shock when fastening on the wires from the lamp to the sledge, although the switch on the wall was turned off. This is easy of explanation, for the switch only cuts off one wire, and the remaining one carried the current to earth through the nurse. Careful attention to details of this kind is necessary, if unpleasant results are to be avoided, and especially should no patient be allowed to get into a bath alone or before it has been tested by dipping both hands into it as far apart as possible, the maximum current to be used having been first turned on. If all is satisfactory the current should then be turned down for the patient to get in and slowly raised afterwards. No patient should be left alone when taking a bath.

[1] Archives d'Electrotherapie.

(*To be continued.*)

